# Emerging diagnostic methods and imaging modalities in cushing’s syndrome

**DOI:** 10.3389/fendo.2023.1230447

**Published:** 2023-07-25

**Authors:** Kyla Wright, Elisabeth F. C. van Rossum, Elcin Zan, Nicole Werner, Alan Harris, Richard A. Feelders, Nidhi Agrawal

**Affiliations:** ^1^ New York University (NYU) Grossman School of Medicine, New York, NY, United States; ^2^ Department of Internal Medicine, Division of Endocrinology, Erasmus Medical College (MC), University Medical Center Rotterdam, Rotterdam, Netherlands; ^3^ Department of Radiology, Weill Cornell Medicine, New York, NY, United States; ^4^ Department of Medicine, Division of Endocrinology, New York University (NYU) Langone Medical Center, New York, NY, United States

**Keywords:** pituitary, cushing’s syndrome, cushing’s disease, hypercortisolemia, diagnosis pseudo-cushing’s states

## Abstract

Endogenous Cushing’s syndrome (CS) is a rare disease characterized by prolonged glucocorticoid excess. Timely diagnosis is critical to allow prompt treatment and limit long-term disease morbidity and risk for mortality. Traditional biochemical diagnostic modalities each have limitations and sensitivities and specificities that vary significantly with diagnostic cutoff values. Biochemical evaluation is particularly complex in patients whose hypercortisolemia fluctuates daily, often requiring repetition of tests to confirm or exclude disease, and when delineating CS from physiologic, nonneoplastic states of hypercortisolism. Lastly, traditional pituitary MRI may be negative in up to 60% of patients with adrenocorticotropic hormone (ACTH)-secreting pituitary adenomas (termed “Cushing’s disease” [CD]) whereas false positive pituitary MRI findings may exist in patients with ectopic ACTH secretion. Thus, differentiating CD from ectopic ACTH secretion may necessitate dynamic testing or even invasive procedures such as bilateral inferior petrosal sinus sampling. Newer methods may relieve some of the diagnostic uncertainty in CS, providing a more definitive diagnosis prior to subjecting patients to additional imaging or invasive procedures. For example, a novel method of cortisol measurement in patients with CS is scalp hair analysis, a non-invasive method yielding cortisol and cortisone values representing long-term glucocorticoid exposure of the past months. Hair cortisol and cortisone have both shown to differentiate between CS patients and controls with a high sensitivity and specificity. Moreover, advances in imaging techniques may enhance detection of ACTH-secreting pituitary adenomas. While conventional pituitary MRI may fail to identify microadenomas in patients with CD, high-resolution 3T-MRI with 3D-spoiled gradient-echo sequence has thinner sections and superior soft-tissue contrast that can detect adenomas as small as 2 mm. Similarly, functional imaging may improve the identification of ACTH-secreting adenomas noninvasively; Gallium-68-tagged corticotropin-releasing hormone (CRH) combined with PET-CT can be used to detect CRH receptors, which are upregulated on corticotroph adenomas. This technique can delineate functionality of adenomas in patients with CD from patients with ectopic ACTH secretion and false positive pituitary lesions on MRI. Here, we review emerging methods and imaging modalities for the diagnosis of CS, discussing their diagnostic accuracy, strengths and limitations, and applicability to clinical practice.

## Introduction

Endogenous Cushing’s syndrome (CS) is characterized by prolonged glucocorticoid excess. Most cases are iatrogenic, resulting from prolonged or high-dose exposure to exogenous glucorticoids (1, 2). Conversely, endogenous CS is a rare disease with an estimated incidence of 0.7-2.4 million per year (3, 4). It is characterized by excessive adrenal cortisol secretion that can be either dependent or independent of adrenocorticotropic hormone (ACTH) secretion. Most cases of CS are ACTH-dependent (80-85%) (5), the causes of which include ACTH-secreting pituitary adenomas and ectopic ACTH or corticotropin-releasing hormone (CRH) secretion (6). ACTH-secreting pituitary adenoma, referred to as “Cushing’s disease (CD),” is the most frequent cause of endogenous CS (7). ACTH-independent Cushing’s syndrome is even rarer (20%), resulting from hypersecretion of cortisol by adrenal pathologies such as adrenal adenomas (6).

Recognizing CS is difficult as many clinical features are non-discriminatory and common in the general population, such as central obesity, weight gain, fatigue, depression, acne, decreased libido, myopathy, and oligo- or amenorrhea (3). However, early diagnosis is critical to mitigate effects of CS on associated comorbidities including metabolic syndrome, cardiac disease, hypercoagulability, osteoporosis, and increased susceptibility to infection (8). In fact, it has been estimated that most CS patients have a >20% risk of a major cardiovascular event within the next 10 years (9) and the mortality rate in these patients is estimated to be four times higher than expected in the general population (10). Thus, timely diagnosis allows prompt treatment and limits long-term disease morbidity and risk of mortality.

To diagnosis CS, current Endocrine Society guidelines recommend biochemical testing with 24-hour urinary free cortisol, low-dose dexamethasone-suppression test or late-night plasma or salivary cortisol, with two of three positive results indicating a diagnosis of CS (11). Once the diagnosis is confirmed, plasma ACTH concentration is measured to differentiate ACTH-dependent and ACTH-independent CS, with elevated plasma ACTH concentrations suggesting ACTH-dependent CS and suppressed plasma ACTH concentrations indicating ACTH-independent CS (12). Because ACTH-dependent CS most commonly originates from corticotroph pituitary adenomas, elevated ACTH levels are typically followed by pituitary magnetic resonance imaging (MRI) (5).

However, several challenges complicate the diagnosis of CS. Traditional biochemical diagnostic modalities each have their limitations and biochemical evaluation is particularly complex in patients whose hypercortisolemia fluctuates daily or occurs in specific episodes varying from days to weeks to even years (termed “cyclic Cushing’s syndrome”) or when delineating CS from physiologic, nonneoplastic states of hypercortisolism (also referred to as “pseudo-Cushing’s syndrome”). Moreover, diagnostic evaluation of suspected CD may be complicated by both false positive and false negative pituitary imaging findings, necessitating dynamic testing or even invasive procedures such as bilateral inferior petrosal sinus sampling (BIPSS) (3, 13). Here, we provide a brief review of the limitations of current diagnostic modalities for the diagnosis of CS. Moreover, we review emerging biochemical methods and imaging modalities for the diagnosis of CS, discussing their diagnostic accuracy, strengths and limitations, and clinical applicability.

## Limitations of traditional diagnostic modalities

Each biochemical test to establish endogenous hypercortisolism has caveats which are important to be aware of when interpreting test results (**Table 1**) (14–17). Urinary free cortisol (UFC) excretion has a limited sensitivity which is in part due to day-to-day variation in cortisol excretion in some patients with CS in whom days with elevated cortisol production alternate with days with normal cortisol excretion (19). Therefore, repeated UFC measurements are often necessary. In addition, UFC measurements can be influenced by gender, age, urinary volume (low, high) and sodium intake (13, 20).

**Table 1:** Diagnostic accuracy and limitations of traditional first-line biochemical tests, as well as the newer hair tests used for the diagnosis of Cushing’s syndrome (14–18).

Test	Sensitivity	Specificity	Disadvantages
Low-Dose Dexamethasone Suppression Test	>95%	80-85%	• Variation in absorption and metabolism of dexamethasone (e.g., P450 enzyme system interactions, liver or renal disease) between patients may influence results • False positives in women taking oral contraceptive pills and during pregnancy due to increased cortisol binding globulin (CBG) • Poor performance in differentiating from nonneoplastic hypercortisolism • Incompliance may cause a false-positive result • No possibility to test cortisol levels in retrospect as is useful in cyclic CS
Late Night Salivary Cortisol	90-98%	90-100%	• False positives in patients who smoke or use chewing tobacco, and with direct contamination of the saliva with exogenous steroids • Influenced by abnormal sleep-wake cycles • False positives in patients of older age and those with hypertension and diabetes mellitus • False positives in foods that contain glycyrrhizic acid (e.g., licorice, teas) • No possibility to test cortisol levels in retrospect as is useful in cyclic CS
24 Hour Urine Free Cortisol	70-75%	40-90%	• Potential for improper collection • Day-to-day intra-patient variability, often requiring repeated measurements • Possible contamination • Poor performance in differentiating from nonneoplastic hypercortisolism • Measurements influenced by gender, age, urinary volume, sodium intake, and renal function • No possibility to test cortisol levels in retrospect as is useful in cyclic CS
Hair Cortisol	81%	88%	• Limited if no or little scalp hair is present • Method is not yet widely available and, therefore, samples often need mailing to expert laboratories
Hair Cortisone	87%	90%	• Limited if no or little scalp hair is present • Method is not yet widely available and, therefore, samples often need mailing to expert laboratories

The low-dose (1 mg) dexamethasone suppression test (LDST) has a high sensitivity to confirm CS. However, incompliance can be a cause of a false-positive result. For this reason, it can be useful to measure plasma dexamethasone concentrations concurrently, which can be measured *via* a rapid, simple, and sensitive liquid chromatography/tandem mass spectrometry (LC–MS/MS) assay (21, 22). Further, overnight dexamethasone suppression testing requires that patients attend a health care facility for venopuncture; however, a recent study reported that salivary cortisone correlates strongly with serum cortisol in the LDST and may be used as an alternative sampling method that can be collected at the patient’s home (23). Additionally, drugs that enhance cortisol-globulin production like estrogens and mitotane can cause higher measured cortisol levels (13, 20) and drugs that interfere with CYP3A4, which metabolizes dexamethasone in the liver, can influence DST results. Inducers of CYP3A4 (e.g., antiepileptic drugs) can cause false-positive results *via* acceleration of dexamethasone breakdown, resulting in less exposure of the pituitary to negative feedback inhibition. Conversely, inhibitors of CYP3A4 (e.g., diltiazem and fluoxetine) can prolong dexamethasone bioavailability, which can suppress ACTH production by adenomas that are sensitive to negative feedback and thus lead to false-negative results (13, 20).

Late night salivary cortisol (LNSC) levels have generally a good performance to establish endogenous hypercortisolism, detecting the failure to achieve a normal circadian nadir in cortisol secretion, and are frequently the first test to become abnormal in patients with recurrent CD (24). While normal individuals typically have a cortisol level < 125 ng/dL during the nadir, higher results support a diagnosis of CS with a sensitivity and specificity of 90-98% and 90-100% respectively (25). That said, cut-off values are assay specific. Caveats include shift working, which disrupts the normal circadian rhythm, and blood contamination. In addition, one study found higher LNSC levels in older subjects without CS, in particular when hypertension and diabetes mellitus were also present (26). Further studies are needed to explore whether the reference values for LNSC should be adjusted according to age and comorbidities. Finally, glycyrrhizic acid, a component of licorice and some teas, inhibits 11beta-hydroxysteroid dehydrogenase type II, which converts cortisol to cortisone (27). Excess use of glycyrrhizic acid can thus lead to elevated salivary cortisol levels.

Limitations of first-line screening tests can further be found in several conditions, including cyclical CS, pregnancy, nonneoplastic states of hypercortisolism, and renal failure. In cyclical CS, episodes of hypercortisolism alternate with periods of biochemical remission. If first-line screening tests are performed in the quite phase, the diagnosis can be missed. Therefore, if cyclical CS is suspected, repeated measurement of UFC and/or LNSC are often necessary to confirm the diagnosis (13).

The diagnosis of CS during pregnancy is challenging because of overlap in clinical features of both conditions and in the physiological changes that occur in hypothalamus-pituitary-adrenal (HPA) axis function. Total plasma cortisol levels start to rise in the first trimester due to an increase in cortisol-binding globulin production and an increase in ACTH secretion. As a result, the LDST can give false positive results (11, 28). UFC levels modestly increase in the second and third trimester and, although UFC levels of more than 3 times the upper limit of normal (ULN) are indicative of CS, it can be difficult to distinguish CS from physiological activation of the HPA-axis when UFC levels are mildly elevated (11, 28). Measurement of LNSC concentrations can be useful to detect CS during pregnancy. LNSC levels physiologically increase during pregnancy, up to two times the ULN in the third trimester, but in CS significantly higher cortisol levels are found (29).

Nonneoplastic states of hypercortisolism are conditions that are accompanied by activation of the HPA-axis like psychiatric disorders, severe obesity, poorly controlled diabetes and chronic alcohol and drug abuse (13, 20). Subjects with nonneoplastic hypercortisolism often have clinical features of CS in combination with increased UFC and/or post-LDST cortisol concentrations (30). LNSC, midnight plasma cortisol levels, and the dexamethasone-CRH test have a good performance to differentiate CS from nonneoplastic hypercortisolism (30). Nevertheless, there is a lack of availability of CRH testing which has limited it’s use in the U.S. Further, some overlap in test results is observed and, in some patients, the diagnostic process can be very challenging.

Diagnosis of CS in a patient with renal failure is difficult because UFC (false-normal due to decreased cortisol clearance) and the LDST (inadequate suppression of plasma cortisol) are not reliable in this condition. Detection of loss of cortisol circadian rhythm by LNSC measurement is the most accurate test to diagnose CS in these patients; however, chronic kidney disease and dialysis can also be accompanied by a disturbed cortisol diurnal rhythm (31).

If ACTH-dependent CS is established, a pituitary cause should be differentiated from an ectopic cause and imaging of the sellar region is the first diagnostic step. Conventional MRI can detect a pituitary adenoma in about 50% of cases, whereas MRI with spoiled gradient recalled

acquisition in the steady state technique has a better diagnostic performance with a detection rate of 80% (32, 33). In patients with non-visible adenomas or adenomas smaller than 6 mm BIPSS is the gold standard to demonstrate a pituitary source of ACTH overproduction (2). Nevertheless, BIPSS has pitfalls which can lead to false-negative results (e.g., catheter malposition, anatomical abnormalities of the inferior petrosal sinus, adenomas unresponsive to CRH) and false-positive results (e.g., IPSS in remission phase of cyclical CS and ectopic CRH production) (34). Noninvasive dynamic tests to differentiate between pituitary-dependent CS and ectopic ACTH production include the CRH test, the high-dose dexamethasone suppression test and the desmopressin test. Although these tests can be useful in a subset of patients, the diagnostic accuracy is lower compared to BIPSS due to overlap in response in both patient groups and discordant test results in individual patients (2, 13).

## Hair cortisol and cortisone

One of the emerging methods to determine hypercortisolism is hair analysis of cortisol and cortisone. It has been shown that glucocorticoids are incorporated in scalp hair. Hair cortisol concentrations (HCC) have been shown to represent long-term systemic exposure to cortisol. In recent years, both hair cortisol and the inactive form, hair cortisone, have been shown to be useful measures to accurately diagnose overt CS (18, 35). Since scalp hair is growing roughly one centimeter a month, levels of cortisol in hair can be used as a retrospective biomarker for long-term cortisol exposure. While standard first-line screening tests capture cortisol exposure at one timepoint or (for) up to several days, hair measurements allow assessment of glucocorticoid concentrations in the past months to years.

The hair test is appealing since the method is non-invasive and sample collection is easy and patient friendly. Standard tests such as a dexamethasone suppression test, collection of 24-hour urine, and consecutive saliva collections can be quite burdensome for patients. In contrast, collection of a small hair sample (with scissors) is quick, is independent of the time of the day (e.g., can easily be performed at the outpatient department), and is not influenced by (non)fasting states, acute stress, test adherence, renal function, and use of drugs which could alter dexamethasone clearance and/or levels of cortisol-binding globulin. The number of laboratories providing hair analysis is increasing, although this method is not yet widely available. Since hair samples can be easily kept at room temperature and kept for at least months, they can easily be sent by mail to laboratories with expertise in hair analysis.

Multiple studies in the field of stress-related research used HCC as a long-term stress biomarker (36). Also, in normal conditions, hair cortisol has been evaluated and compared to more commonly used cortisol measures. Short et al. performed a validation study in a small sample of healthy participants to investigate the extent to which HCC captures cumulative cortisol production (37). They examined the correspondence of HCC obtained from the proximal 1 cm hair segment with 30-day mean salivary cortisol area-under-the curve (AUC) based on three samples collected per day (on awakening, +30 min, and at bedtime) as well as the mean of four weekly 24-h UFC assessments. They found that HCC was most strongly associated with the prior 30-day integrated cortisol production measure (as estimated by average salivary cortisol AUC) (r=0.61, p=0.01). Their findings support the notion that HCC can be used as a reliable estimate of long-term integrated free cortisol production that is corresponding to integrated salivary cortisol production measured over a one-month period. Cross-sectional population-based studies showed consistent positive associations between hair glucocorticoids and BMI as well as waist circumference, as would be expected due to the known glucocorticoid tissue effects (38). Interestingly, the most prominent and clinically relevant associations were observed for hair cortisone and waist circumference.

In the past decade, the clinical relevance of hair glucocorticoid measurements in the field of CS diagnostics has become evident (18). Hair cortisol, but even stronger, hair cortisone, has been shown to accurately differentiate with high sensitivity and specificity between healthy controls and CS patients (18, 39). HCC have been shown to correlate significantly with UFC within patients with CS (35). Most studies use a 3 cm hair fragment per individual patient. Hair glucocorticoid measurements are performed with either immunoassay (35, 40) or liquid chromatography tandem mass spectrometry (LC-MS/MS) (18, 39). The glucocorticoid analysis in the 3 cm hair segment corresponds to mean glucocorticoid levels of approximately the past 3 months. In accordance with the observed strong correlations between hair cortisone and waist circumference in population-based studies (38), we also found that in CS patients, hair cortisone has a higher differentiating capacity (sensitivity 87%, specificity 90%) than hair cortisol (sensitivity 81%, specificity 88%) (**Table 1**) (18).

An important issue in clinical practice is the endocrine evaluation of adrenal incidentalomas (41). These incidentally found, mostly benign adenomas can secrete cortisol autonomously, which is often a mild hypersecretion in which a typical CS phenotype is lacking. This condition has been known as subclinical Cushings (42, 43) and has been renamed mild autonomous cortisol secretion (MACS) or possible ACS (44) (41). Since these chronically, slightly elevated cortisol levels seem to be associated with a variety of cardiometabolic comorbidities (45–47) and increased mortality (48–50), it is relevant to detect the hypercortisolism and to quantify it. In fact, a large study from the European Network for the Study of Adrenal Tumors found that all-cause mortality is particularly high in adult females less than 65 years of age with autonomous cortisol secretion, whereas mortality was only moderately increased in women >65 and men <65 years of age, and was not affected in men >65 years of age (51).

The main diagnostic tool to evaluate adrenal incidentalomas is the 1-mg overnight dexamethasone suppression test (DST), with cortisol levels ≤ 50 nmol/L excluding ACS, cortisol levels > 138 nmol/L indicating ACS, and cortisol levels between 50 and 138 nmol/L defined as possible ACS (52). However, the DST can yield false positive or negative test results. Also, measurement of UFC, midnight salivary cortisol, and morning plasma ACTH can contribute to the diagnosis, but their value has been shown to be limited due to their only short-term or timepoint measurement as well as possible interferences and analytical issues (53).

It would make sense that hair glucocorticoids could fill this diagnostic gap, as chronic subtle glucocorticoid excess could be reflected in hair. Indeed, Brossaud et al. reported higher hair cortisol and cortisone levels in mild CS compared to healthy controls (p < 0.01) (39). They also showed that the performance of hair cortisol and hair cortisone to diagnose overt CS was even better (92 and 100% sensitivity, 91 and 99% specificity, respectively). The sensitivity and specificity to diagnose mild CS was lower compared to overt CS for both hair cortisol and hair cortisone (sensitivity: 59% and 68%, and specificity: 79 and 94%, respectively). In accordance with the better performance of hair cortisone in studies with ACTH-dependent CS as well as features in population-based studies, hair cortisone was correlated with midnight serum cortisol (p < 0.02) and volume of adrenal incidentalomas (p < 0.04). Hair cortisone did not correlate with UFC. However, in contrast to the normal UFC, both hair cortisol and cortisone were in the range of overt CS in 11 out of 23 patients with mild CS. Interestingly, patients with mild CS and increased hair cortisone required more antihypertensive treatments and showed more unfavorable lipid profiles than patients with normal hair cortisone. Thus, Brossaud et al. demonstrated that hair glucocorticoid measurement performed better in overt than in mild CS. They concluded that hair steroid analysis is a useful adjunct to diagnose mild CS and to identify adrenocortical incidentalomas excessively secreting cortisol.

These findings were not confirmed in a recent study of Puglisi et al. who analyzed data of 67 adrenal incidentaloma patients (of whom 32 patients had autonomous cortisol production) and 81 healthy subjects (54). No differences in hair cortisol or cortisone concentrations between healthy controls and adrenal incidentaloma patients were found. In addition, no significant difference was found in hair cortisol and cortisone in adrenal incidentaloma patients with or without autonomous cortisol production (definition based on serum cortisol after 1 mg dexamethasone). It should be noted that in the latter study, most patients had normal levels of 24-hour UFC and plasma ACTH was suppressed only in few patients, indicating that probably only a minimal degree of cortisol excess must have been present.

Another clinically relevant aspect of hair glucocorticoid analysis is that scalp hair can be used as a historical timeline. In particular, hair analysis can be useful in detecting cyclic CS (40, 55). The cyclical variant of CS is often a diagnostic challenge, since these patients periodically secrete excess cortisol yielding normal test results when patients are screened at a moment between episodes of hypercortisolism. We previously reported in a set of patients suspected of cyclic Cushing’s syndrome that retrospective timelines of hair cortisol indeed showed that dynamic cortisol concentrations over time corresponded well with clinical cushingoid features (40).

An additional, albeit rare, condition for which hair analysis can be valuable to detect retrospective hypercortisolism is pituitary apoplexy. In this situation, the apoplexy may induce acute remission of the hypercortisolism that was due to an ACTH overproducing adenoma, while many features of the clinical phenotype (e.g., increased abdominal fat mass, muscle atrophy, decreased bone mineral density) may persist. In these cases, the diagnosis cannot biochemically be confirmed by routine tests for Cushing’s syndrome. This was demonstrated in a case with an evident clinical picture of Cushing’s syndrome, who presented with pituitary apoplexy (56). Hair cortisol analysis clearly showed hypercortisolism in the period before the apoplexy. This is of clinical importance since clinicians may anticipate the sequelae of the previous extended period of severe hypercortisolism, such as symptoms of (relative) hypocortisolism afterwards, as well long-lasting mental and physical complaints after remission of CS (40).

In summary, scalp-hair cortisol and cortisone measurements are emerging valuable tests to assess long-term glucocorticoid exposure in patients with CS. Also, hair glucocorticoid analysis is a valuable addition to the current limited options to diagnose transient periods of hypercortisolism in retrospect as present in cyclic CS or pituitary apoplexia which was preceded by a clinical picture of CS.

## The desmopressin test

Desmopressin is a synthetic analogue of arginine vasopressin (AVP). AVP is an important regulator of ACTH secretion, acting as an ACTH secretagogue at the pituitary vasopressin receptor (receptor V3) (57). In contrast, desmopressin is selective for the renal AVP receptor (receptor V2), resulting in an antidiuretic effect that makes it the treatment of choice for central diabetes insipidus (58). In most healthy adults, desmopressin’s effect on ACTH secretion is negligible (57). In contrast, patients with ACTH-dependent CS exhibit an enhanced ACTH and cortisol response following administration of desmopressin (59, 60).

The cortisol responses to desmopressin in CS was first evaluated in 1993 by Malerbi et al., finding that a cortisol increase by 40-45% from baseline was seen in all but one of the 16 patients with CD, but was not observed in the patients with adrenal CS (59). Thereafter, the desmopressin test became widely adopted across Europe, but it’s utilization in the United States is relatively recent. Though the mechanism of this abnormal response is not fully elucidated, aberrant expression of the renal V2 receptor on corticotroph tumors may be responsible (61). Consequently, a desmopressin (DDAVP) stimulation test may be utilized in the diagnosis of CS.

The desmopressin stimulation test is performed by measuring plasma ACTH and serum cortisol levels before and after 10 μg of DDAVP administration. In general, a cortisol rise >20% above basal and an ACTH rise >50% above basal supports a diagnosis of CS; however, cut-off values are not universally accepted and vary significantly within the literature (62). Because this aberrant desmopressin response is a result of neoplastic cells, it is particularly useful for distinguishing patients with endogenous CS from nonneoplastic hypercortisolism (57). In fact, the desmopressin stimulation test has an estimated sensitivity and specificity to distinguish endogenous CS from nonneoplastic hypercortisolism of 75-90% and 90-92% respectively (16). A recent meta-analysis by Mondin et al. found that the desmopressin test had high specificity (>90%) for differentiating patients with nonneoplastic hypercortisolism from those with CS (63). Notably, the specificity may be lower than the CRH test and the CRH stimulation test after dexamethasone suppression (16, 63, 64), but it may be a simpler and more widely available diagnostic test.

Further, diagnostic accuracies reported in the literature vary by cut-off values. Using a peak ACTH level >6 pmol/L (>27 pg/mL), Moro et al. identifying 90% of patients with CD with a specificity of 96.7% (64). With this same cut-off value, Pecori et al. reported a sensitivity of 81% and specificity of 90% (65), and Tirabassi et al. reported a sensitivity of 75% and specificity of 89% (66). Interestingly, when Tirabassi et al. used a baseline serum cortisol >12 ug/dL and peak ACTH level >4 pmol/L (>18.18 pg/mL), their sensitivity and specificity increased to 90% and 91% respectively % (66). Lastly, a recent study by Rollin et al. found that using a cut-off peak ACTH value of >15.8 pmol/L (>71.8 pg/ml), they were able to diagnose CD with a sensitivity of 90.8% and specificity of 94.6%, yielding a positive and negative predictive value of 95.3% and 89.9% respectively (67).

The utility of the desmopressin test has also been investigated in the setting of ACTH-dependent CS. In the initial study by Malerbi et al., the three patients with suspected ectopic-ACTH secretion did not exhibit a positive desmopressin test (59). This theory was also supported in early studies (68, 69); however, subsequent studies have demonstrated that patients with CS secondary to confirmed ectopic-ACTH secretion may exhibit positive cortisol responses as well (60, 70), suggesting that the desmopressin test may have a limited role in differentiating ACTH-dependent CS from CD (57). In fact, studies report between 0-75% of ectopic ACTH-secreting lesions will show stimulation with desmopressin (57).

Conversely, a recent meta-analysis comparing three dynamic testing methods (including the CRH test, the desmopressin test, and the high-dose dexamethasone suppression test [HDDST]) found that the desmopressin test, though it was reported on in fewer studies, had a high sensitivity for CD (up to 85%) (71). Still, the CRH test had the highest sensitivity for detecting CD (ACTH 86.9%, cortisol 86.2%) and highest specificity for detecting ectopic-ACTH secretion (ACTH 93.9%, cortisol 89.4%) (71). Thus, the CRH test appears to differentiate CD and ectopic ACTH secretion with the greatest diagnostic accuracy but when considering the shortage of CRH in the United States, the desmopressin test may be useful when CRH is not readily available.

The role of the desmopressin test in assessing postoperative remission and recurrence has also been of interest. Transsphenoidal resection, the most efficacious therapeutic option for patients with CD, results in remission in 70-90% of patients. Still, recurrence has been reported to occur in up to 66% of patients during follow-up (72). The desmopressin test has been suggested to serve as a prognostic marker of long-term risk of recurrence in the immediate post-operative period as well as a useful early marker of recurrence during long-term follow-up (57). Many biochemical tests have been suggested as postoperative predictors of long-term outcomes, such as a very low postoperative cortisol level (73) or a normal dexamethasone suppression test (74). Though a very low early postoperative cortisol level is the most widely adopted, none of the current markers can predict long-term remission with high accuracy (57, 72).

Unlike other proposed markers, the desmopressin test is able detect the presence of residual neoplastic corticotrophs and thus risk of future recurrence. Prior studies report sensitivities ranging from 20-100% and specificities between 57-100% (57). That said, there is significant variation between definitions of desmopressin response, remission, and recurrence, and follow-up period between studies. Many studies defined a positive desmopressin response by relative increases in cortisol or ACTH, leaving room for false positives in patients with very low postoperative cortisol levels that exhibited small variations in cortisol levels in response to dexamethasone. Instead, using an absolute cortisol increment may improve diagnostic accuracy, resulting in a specificity and sensitivity of 95% and 68% respectively (57, 75–78). One study by Vassiliadi et al. found that an increase in serum cortisol ≥7.4 μg/dL from baseline following desmopressin administration had a hazard ratio for recurrence of 24.7 (78) – superior to the more commonly used criterion of very low or undetectable cortisol levels in the immediate postoperative period. Thus, the desmopressin test may augment our ability to assess a patient’s long-term risk of recurrence when used in the immediate postoperative period, with a loss of the paradoxical response indicating a more favorable outcome. However, larger prospective studies with longer follow-up periods are needed.

Furthermore, detecting early recurrence remains a challenge in the postoperative follow-up of CD patients. In the early recurrence periods, there is low disease activity that may render traditional biochemical tests only marginally abnormal. Earlier detection of recurrence allows for more timely intervention and minimizes risk of prolonged hypercortisolemia. One patient series reported by Ambrosi et al. described three patients who, though initially had negative postoperative desmopressin tests, later exhibited positive desmopressin tests during follow-up. In all three patients, the positive desmopressin test preceded other biochemical abnormalities indicating recurrence by 4-39 months (79). Similarly, another study found that 17 of 20 patients with recurrent CD had prior positive desmopressin tests that preceded an increase in midnight cortisol or UFC in 71% of patients (80). Lastly, a recent study reported that, during long-term follow-up of 43 CD patients in remission following transsphenoidal resection, all patients with initially negative desmopressin tests that later had positive responses during follow-up experienced recurrence. In these patients, the positive desmopressin tests preceded frank recurrence by years (81). Thus, a positive desmopressin test may serve as an earlier marker of recurrence in the follow-up of CD patients achieving remission following transsphenoidal resection.

## Combination tests

For patients with ACTH-depending Cushing’s syndrome, determining the origin of ACTH secretion can be challenging. Although there are many diagnostic tests, the overlapping biochemical features between CD and ectopic ACTH syndrome (EAS) prevent any one biochemical test from exhibiting complete specificity. The most commonly used algorithm utilizes a 2-step strategy in which dynamic biological testing is evaluated in combination with a pituitary MRI (82). In general, if there is no evidence of a pituitary adenoma > 6 mm in diameter on MR imaging or if the results of biochemical testing are inconclusive, BIPSS may be performed. Another strategy, used in specialized centers, is to directly perform BIPSS, considering the limitations of dynamic tests. BIPSS is considered the “gold standard” diagnostic procedure for identifying CD, as it has a sensitivity and specificity of 94% (83). Still, the diagnostic test has limitations: BIPSS is an invasive procedure with associated risks and its availability varies. Moreover, false negatives may occur in individuals with variations in the venous anatomy of the petrosal sinuses and in patients with cyclical CS (83, 84). As such, noninvasive diagnostic strategies that combine multiple biochemical analyses with imaging studies have been of interest and can reduce the need for BIPSS in a subset of patients (83). The diagnostic accuracies of such strategies are summarized in **Table 2** (33, 83, 85–87).

**Table 2:** Summary of non-invasive strategies and reported diagnostic accuracies for differentiating Cushing’s Disease and ectopic ACTH syndrome (33, 83, 85–87).

Strategy	Positive Result Suggestive of CD	Sensitivity	Specificity
Individual tests			
CRH Stimulation Test (83)	• >35% increase in ACTH concentration after CRH administration	90%	90%
High-Dose Dexamethasone Suppression Test (85)	• >50% suppression of cortisol concentrations	81%	66.70%
Pituitary MRI (33)	• Lesion compatible with a pituitary adenoma identified on standard MRI by two experienced radiologists	46-49%	33-50%
Combination tests			
CRH Stimulation Test + Desmopressin Stimulation Test (86)	1. >17% increase in cortisol and >37% increase in ACTH following CRH stimulation 2. >18% increase in cortisol and >33% increase in ACTH following desmopressin stimulation	73%	93%
CRH Stimulation Test + Desmopressin Stimulation Test + Pituitary MRI (86)	1. >17% increase in cortisol and >37% increase in ACTH following CRH stimulation 2. >18% increase in cortisol and >33% increase in ACTH following desmopressin stimulation 3. Pituitary lesion compatible with a pituitary adenoma on MRI	49.70%	100%
CRH Stimulation Test + High-Dose Dexamethasone Suppression Test (87)	1. >72% increase in ACTH following CRH administration 2. >52.7% decrease in cortisol following dexamethasone administration	75.60%	100%

*CD*, Cushing’s Disease; *CRH*, corticotropin releasing hormone; *ACTH*, adrenocorticotropic hormone; *MRI*, magnetic resonance imaging.

A recent study by Frete et al. investigated the diagnostic utility of combining the CRH and desmopressin stimulation tests with pituitary MR imaging to differentiate CD from EAS, finding that CD was detected with greater sensitivity and specificity than either test in isolation. In this analysis, a relative increase in cortisol >17% and an increase in ACTH > 37% in response to CRH stimulation was considered indicative of CD. A desmopressin stimulation test yielding a relative cortisol increase >18% and ACTH increase >33% was considered consistent with CD. All patients had a pituitary MRI. In cases in which biochemical and imaging results were discordant, or if the tests were indicative of EAS, a thin-slice whole body computed tomography (CT) scan was performed prior to BIPSS. When analyzed in isolation, the CRH stimulation test exhibited a sensitivity and specificity of 83%. The desmopressin stimulation test demonstrated a sensitivity of 85% and specificity of 81%. Combining the results of the two biochemical assays, the specificity increased to 93% with a positive predictive value (PPV) of 98%. Incorporating the results of imaging studies improved the PPV further: the PPV for CD was 100% in patients with positive responses to the CRH and desmopressin tests who had MRI findings indicative of a pituitary adenoma. Similarly, there was a 100% PPV for CD in patients whose CRH and desmopressin tests yielded positive responses and whose imaging studies showed a negative pituitary MRI and negative whole-body CT. In addition, there was a 100% negative predictive value (NPP) for CD in patients with negative responses to the CRH and desmopressin tests, negative pituitary MRI, and a CT scan positive for EAS. Thus, in these groups, BIPSS becomes unnecessary, allowing the procedure to be avoided in 47% of patients with ACTH-dependent Cushing’s syndrome (86).

Another study by Barbot et al. found that combining the results of a patient’s CRH stimulation test and dexamethasone 8 mg overnight suppression test (HDDST) allowed clinicians to distinguish CD from EAS more reliably. In this study, a CRH stimulation resulting in an increase in ACTH >72% and a HDDST resulting in a decrease in cortisol by >52.7% were considered positive for CD. When the results of these two tests were assessed in series, two positive tests were 75.6% sensitive for diagnosing CD and two negative tests were 100% sensitive for identifying EAS. None of the analyzed cases of EAS yielded positive results in both tests and 5.6% of cases of CD demonstrated negative results in both tests. The authors suggested that by combining the CRH stimulation test with the HDDST, patients with a positive result in both tests may not require BIPSS (87).

Lastly, concordant positive responses to dynamic testing may be sufficient to diagnose CD, irrespective of MRI findings. A recent study by Ferrante et al. found that, among patients with negative pituitary MR imaging (undetectable or detectable lesion <6 mm), a positive response to the CRH test, HDDST, and desmopressin test was present in 89.4% of patients. A concordant positive response to both the CRH test and HDDST or the CRH test and desmopressin test had a positive predictive value of 100% for the diagnosis of CD. Thus, the authors suggest that, even in patients with negative pituitary MRI findings, concordant positive response to noninvasive dynamic tests is sufficient to diagnose CD and that BIPPS can be reserved for cases in which responses are discordant (88).

## Advances in imaging techniques

The available non-invasive diagnostic tests for CS are divided into two major categories based on *anatomical* imaging, which provides morphological information, and *molecular positron emission tomography (PET)* imaging, which provides functional and morphological information when combined with CT or MRI.

Anatomical imaging modalities, including CT and MRI, rely on high resolution and intravenous contrast information, yielding differential enhancement between native glands (pituitary or adrenal) and the adenomas. The detection rates are higher in lesions larger than 1 cm, but there is a chance that these diagnostic tests may fail if the lesions are too small or do not reveal discrete contrast information due to aberrant vascularity. MRI has inherently high spatial and soft tissue contrast compared to CT and is the workforce for pituitary adenomas. In adrenal gland imaging, CT is commonly preferred over MRI due to wide availability, and fast acquisition.

A standard pituitary MRI protocol on a 3T magnet includes dynamic T1W image acquisition which has a limitation of 6 mm for minimal lesion size detection. Conversely, postcontrast Golden-Angle Radial Sparse Parallel (89) sequencing obtained on a 3T magnet is well suited to achieve submillimetric (aproximately 0.8 mm) isotropic voxel resolution during a 180- second scan timeframe (90). Temporal resolution is reconstructed to 20 seconds per frame, allowing for the dynamic view of contrast information. Besides the qualitative evaluation, quantitative analysis can be achieved by postprocessing the permeability maps extracted from the GRASP data. A region-of-interest (ROI) is drawn to generate corresponding time-dependent enhancement curves, differentiating microadenomas from non-functional cysts or macroadenomas from hyperplasia of the gland. Despite all the advancement in pituitary MRI, up to 40% of cases are “MR imaging–negative”. A further consequence of this is the unknown CD-free prognosis in those patients, even if further diagnostic studies such as BIPPS are carried-out and lead to surgical operation (91).

Contrast-enhanced MRI has a sensitivity of 50% to 60% and a PPV of 86% for detecting adrenocorticotropin-secreting pituitary tumors (33). In up to 90% of cases, CD is caused by microcorticotropinoma, with the majority being less than 6 mm in size, which could be a limitation of MRI tissue resolution. Further, MRI cannot distinguish non-functional pituitary adenomas from functional adenomas in the case of solid enhancement. In MRI negative CD, BIPSS has excellent accuracy in differentiating ectopic from a pituitary source of ACTH production, but the PPV for corticotropinoma lateralization is 60% - 70% (92), leading to only 45% curative rates (93). In fact, with the currently available advanced diagnostics, there is a 30% to 40% risk of failure to detect a pituitary microadenoma, negatively effecting the intraoperative yield for selective pituitary adenomectomy.

Moreover, with innovations in PET detector technology and image reconstruction algorithms, there have been an increasing number of reports using PET for pituitary adenoma. Molecular PET imaging is well-suited to address the shortcomings of MRI and BIPSS, providing highly sensitive functional information. Molecular PET can be performed as PET/CT or PET/MRI. The latter is preferred for pituitary gland evaluation, as it provides volumetric MRI coregisteration, achieving subcentimeter tissue resolution and excellent diagnostic lesion localization. Though earlier studies investigated the detection of pituitary adenomas using ^18^F-fluorodeoxy-glucose (FDG), pituitary FDG-uptake was shown to be dependent on secretory activity, leading to diurnal variation and reduced sensitivity (94). More recent studies have primarily focused on using amino acid analogues such as ^11^C-Methionine (MET) and ^18^F-Fluoroethyl-L-tyrosine (FET) or ^68^Ga-Corticotrophin Releasing Hormone (CRH) as suitable radiopharmaceuticals.

Aside from practical differences, both methionine and fluoroethyltyrosine are taken up at sites of peptide synthesis, possibly *via* the same l-type amino acid transporter (LAT1). Early studies confirmed increased ^11^C-MET uptake in most types of pituitary adenomas and several groups have demonstrated the utility of MET PET/MRI to localize small corticotroph adenomas (95). However, ^11^C-MET PET has a significant limitation of a short half-life (~ 20 min), which necessitates on-site cyclotron availability. In contrast, ^18^F-FET PET with its longer half-life (~ 110 min) can be synthesized and then transported to off-site centers. Though only small cohorts of patients have been studied with these radiopharmaceuticals, the sensitivity and specificity for FET-PET/MRI for tumor localization was 100% (95% CI 66.37-100%) with histopathology comparison (95, 96). Further, somatostatin-based radiotracers such as ^68^Ga-DOTATATE combined with PET/CT or PET/MRI improves the detection and localization of ACTH-secreting tumors, and may be particularly useful for localizing ectopic ACTH-secreting lesions (97) (98)

CRH is the major regulator of ACTH secretion from the adenohyphysis and acts *via* CRH-1 receptors. The receptor expression status is retained in autonomous corticotropinomas, which is the target for ^68^Ga-CRH PET. In modern PET scanners, the molecular PET are combined with either CT (PET/CT) or MRI (PET/MRI). ^68^Ga- CRH PET/MRI in particular may provide a one-stop shop for functional and morphological assessment of pituitary adenomas.

In a recent study published by Walia et al. (99), ^68^Ga-CRH PET/CT correctly detected corticotropinoma in all 24 cases of CD, all of which were confirmed on histopathology. The advantage of ^68^Ga-CRH PET imaging is that it provides a global view of not only the pituitary but the whole body, including the adrenal glands. It can detect any ectopic ACTH-secreting lesions (e.g., lungs) and adrenal gland abnormalities such as adenomas that may not be detected otherwise. The same authors showed that only 10/24 patients with CD had adenomas < 6 mm in size and in only 4/10 was a lesion not visualized on MRI. Larger validation studies are needed to further assess the value of ^68^Ga-CRH PET imaging beyond MRI-negative or MRI-inconclusive CD. The potential utility in early recurrence, metastatic disease and identifying the mechanisms of lack of treatment response is yet to be elucidated.

Lastly, there is growing interest in using artificial intelligence (AI), specifically machine learning and deep learning algorithms, as a diagnostic tool for imaging analysis to enhance tumor assessment and improve diagnostic accuracy. AI has already demonstrated success in brain tumor MRI radiomic analysis, accurately differentiating between pituitary tumors (100, 101). While several studies have used AI to predict post-operative outcomes in patients with Cushing’s disease (102–104), there is little data on preoperatively differentiating pituitary neuroendocrine tumors using radiomics. Similarly, several studies have used texture analysis to differentiate benign and malignant adrenal lesions (105, 106), but these methods have not yet been applied to identify cortisol hypersecretion in adrenal incidentalomas. Still, this is an interesting area of future study.

## Discussion

The diagnosis of endogenous CS is fraught with challenges, including differentiating it from related conditions such as nonneoplastic hypercortisolism and accurately making the diagnosis in patients with cyclic hypercortisolemia or certain patient populations like pregnant women and those with renal disease. To mitigate these challenges, several new diagnostic methods and imaging modalities have emerged within the literature. Each aims to improve diagnostic accuracy, limit the need for invasive diagnostic tests, and allow for prompt diagnosis and treatment, reducing the impact of CS on related comorbidities and limiting long-term disease morbidity and mortality risk (8, 10).

Emerging modalities such as hair cortisol and cortisone measures and desmopressin stimulation testing may diagnose endogenous CS with higher accuracy, overcoming some of the limitations of traditional biochemical testing. In addition, hair cortisol and cortisone measures are especially useful for the diagnosis cyclic-CS. The desmopressin stimulation test is thought to be particularly useful for differentiating endogenous CS from nonneoplastic hypercortisolism. It has also been suggested to serve as a prognostic marker of long-term risk of recurrence and as a useful early marker of recurrence during long-term follow-up.

However, further efforts are required to improve and standardize the methods for extracting and measuring hair cortisol and cortisone levels, as protocols vary globally. In addition, studies comparing the accuracy of immunoassay and mass spectrometry techniques are needed (107); though immunoassays are relatively easy and inexpensive, they are an indirect measurement and prior studies comparing the two techniques have demonstrated differences in accuracy (108). Interestingly, multiple other steroid hormones can simultaneously be measured in scalp hair using LC-MS/MS based techniques (109). To what extent these steroid profiles may also contribute to the diagnosis of CS or other steroid hormone-related diseases needs further investigation.

Further, there is variability in the reported sensitivity and specificity of the desmopressin stimulation test depending on cut-off values used for peak ACTH and serum cortisol levels. Future studies should examine optimal cut-off values to maximize diagnostic accuracy, improve comparability between studies, and facilitate clearer result interpretation.

After confirming the diagnosis of CS, distinguishing between CD and ectopic ACTH secretion poses distinct challenges, with no single test demonstrating absolute specificity. In effort to improve noninvasive diagnostic accuracy, combining biochemical assays such as the CRH and desmopressin stimulation tests with imaging studies can reduce the need for BIPSS in a subset of patients. Studies have demonstrated high sensitivity and specificity, allowing for the avoidance of BIPSS in up to 47% of patients with ACTH-dependent Cushing’s syndrome (86). However, though combining the desmopressin test with CRH has been previously suggested to improve the test’s ability to diagnose ACTH-dependent CS, the performance of this combined test has not been reliability demonstrated (57).

Further, use of molecular PET imaging for pituitary adenoma may diminish the shortcomings of traditional pituitary MRI and invasive diagnostic methods such as BIPSS. Few, small cohort studies have demonstrated increased ^11^C-MET and ^18^F-FET uptake in pituitary adenomas, which may have utility in detecting small corticotroph adenomas (95, 96). However, uptake of amino-acid analogue radiopharmaceuticals is not specific to corticotropinomas. In contrast, ^68^Ga-CRH PET imaging is specific to ACTH-secreting lesions and allows for both morphological and functional assessment of pituitary adenomas, while also allowing for detection of ectopic ACTH-secreting lesions elsewhere in the body.

While not covered at length in this manuscript, identifying patients to screen for Cushing’s syndrome is a challenging task as the clinical presentation is variable, and many features are commonly found in the general population. Clinical scoring systems have the potential to serve as valuable and straightforward diagnostic tools for assisting in the identification of at-risk patients. Several clinical scores for Cushing’s syndrome have been published, the first of which was developed in 1964 by Nugent et al. (110) but was not widely adopted into clinical practice due to concerns regarding a high number of false negatives. More recent clinical scoring systems have demonstrated improved sensitivity but specificity is not satisfactory, such that patients require additional biochemical testing for diagnostic confirmation or exclusion (111, 112). For example, the “Cushing score” developed by Parasiliti-Caprino et al. aims to distinguish patients with a high- versus low-pretest probability of CS based on baseline clinical features (e.g., facial fullness, hirsutism, obesity, hypertension) with an AUC of 0.87 (sensitivity 96.2%, specificity 82.9%) (112). Given the heterogenous nature of CS, it is unclear if any current clinical scoring model can accurately identify high-risk patients. as recent scoring systems have not been externally validated. Still, they may serve as useful guides, particularly for primary care physician. Additional validation studies in the future could enhance their utility (113, 114).

The emerging diagnostic methods discussed here may help mitigate the limitations of traditional methods used to diagnose CS and improve diagnostic accuracy while reducing burden and risk to patients. Still, many of these methods are relatively new and the diagnostic accuracies and limitations of each have yet to be fully elucidated. Larger, multiinstitutional validating studies are warranted. Further, additional studies to clarify optimal protocols and cut-off values are necessary to improve comparability between studies. Standardization may help improve the ease of which test results are interpreted as well as provide a basis for future societal diagnostic guidelines. Nevertheless, availability of the resources necessary to carry out these novel techniques are likely to vary significantly between institutions and the feasibility and cost-effectiveness of routine implementation of these methods into diagnostic algorithms is unclear.

In conclusion, there are several novel biochemical and imaging techniques that hold potential to transform the diagnostic work-up of CS, addressing many of the short-comings of traditional diagnostic tests. Nonetheless, the diagnosis of CS remains complex. As the diagnostic accuracy and feasibility of these innovative modalities continue to be studied, patients at specialized institutions for whom diagnostic uncertainty persists despite traditional work-up may benefit from the novel diagnostic methods described.

## Author contributions

Authors KW, ER, EZ, NW, AH, and RF contributed to the writing and editing of the manuscript. Author NA contributed to the conceptualization, review, and editing of the manuscript as well as supervised all contributions. All authors contributed to the article and approved the submitted version.

## References

[1] Hopkins RL Leinung MC. Exogenous Cushing's syndrome and glucocorticoid withdrawal. Endocrinol Metab Clin North Am (2005) 34(2):371–84. doi: 10.1016/j.ecl.2005.01.013 15850848

[2] Lacroix A Feelders RA Stratakis CA Nieman LK. Cushing's syndrome. Lancet (2015) 386(9996):913–27. doi: 10.1016/S0140-6736(14)61375-1 26004339

[3] Newell-Price J Bertagna X Grossman AB Nieman LK. Cushing's syndrome. Lancet (2006) 367(9522):1605–17. doi: 10.1016/S0140-6736(06)68699-6 16698415

[4] Sharma ST Nieman LK Feelders RA. Cushing's syndrome: epidemiology and developments in disease management. Clin Epidemiol (2015) 7:281–93. doi: 10.2147/CLEP.S44336 PMC4407747 25945066

[5] Arnaldi G Angeli A Atkinson AB Bertagna X Cavagnini F Chrousos GP. Diagnosis and complications of Cushing's syndrome: a consensus statement. J Clin Endocrinol Metab (2003) 88(12):5593–602. doi: 10.1210/jc.2003-030871 14671138

[6] Tritos NA Biller BM Swearingen B. Management of cushing disease. Nat Rev Endocrinol (2011) 7(5):279–89. doi: 10.1038/nrendo.2011.12 21301487

[7] Raff H Carroll T. Cushing's syndrome: from physiological principles to diagnosis and clinical care. J Physiol (2015) 593(3):493–506. doi: 10.1113/jphysiol.2014.282871 25480800 PMC4324701

[8] Pivonello R Isidori AM De Martino MC Newell-Price J Biller BM Colao A. Complications of Cushing's syndrome: state of the art. Lancet Diabetes Endocrinol (2016) 4(7):611–29. doi: 10.1016/S2213-8587(16)00086-3 27177728

[9] Mancini T Kola B Mantero F Boscaro M Arnaldi G. High cardiovascular risk in patients with Cushing's syndrome according to 1999 WHO/ISH guidelines. Clin Endocrinol (Oxf) (2004) 61(6):768–77. doi: 10.1111/j.1365-2265.2004.02168.x 15579193

[10] Etxabe J Vazquez JA. Morbidity and mortality in Cushing's disease: an epidemiological approach. Clin Endocrinol (Oxf) (1994) 40(4):479–84. doi: 10.1111/j.1365-2265.1994.tb02486.x 8187313

[11] Nieman LK Biller BM Findling JW Newell-Price J Savage MO Stewart PM. The diagnosis of Cushing's syndrome: an Endocrine Society Clinical Practice Guideline. J Clin Endocrinol Metab (2008) 93(5):1526–40. doi: 10.1210/jc.2008-0125 PMC2386281 18334580

[12] Pappachan JM Hariman C Edavalath M Waldron J Hanna FW. Cushing's syndrome: a practical approach to diagnosis and differential diagnoses. J Clin Pathol (2017) 70(4):350–9. doi: 10.1136/jclinpath-2016-203933 28069628

[13] Savas M Mehta S Agrawal N van Rossum EFC Feelders RA. Approach to the patient: diagnosis of cushing syndrome. J Clin Endocrinol Metab (2022) 107(11):3162–74. doi: 10.1210/clinem/dgac492 PMC9681610 36036941

[14] Ceccato F Boscaro M. Cushing's syndrome: screening and diagnosis. High Blood Press Cardiovasc Prev (2016) 23(3):209–15. doi: 10.1007/s40292-016-0153-4 27160717

[15] Nieman LK. Cushing's syndrome: update on signs, symptoms and biochemical screening. Eur J Endocrinol (2015) 173(4):M33–8. doi: 10.1530/EJE-15-0464 PMC4553096 26156970

[16] Findling JW Raff H. DIAGNOSIS OF ENDOCRINE DISEASE: Differentiation of pathologic/neoplastic hypercortisolism (Cushing's syndrome) from physiologic/non-neoplastic hypercortisolism (formerly known as pseudo-Cushing's syndrome). Eur J Endocrinol (2017) 176(5):R205–r16. doi: 10.1530/EJE-16-0946 28179447

[17] Agrawal N Kim H Wright K Mehta S. Hormone excess syndromes of the hypothalamic-pituitary axis. In: Uwaifo GI, editor. The Human Hypothalamus: Anatomy, Dysfunction and Disease Management. Cham: Springer International Publishing (2021). p. 181–213.

[18] Savas M Wester VL de Rijke YB Rubinstein G Zopp S Dorst K. Hair glucocorticoids as a biomarker for endogenous cushing's syndrome: validation in two independent cohorts. Neuroendocrinology (2019) 109(2):171–8. doi: 10.1159/000498886 30759443

[19] Petersenn S Newell-Price J Findling JW Gu F Maldonado M Sen K. High variability in baseline urinary free cortisol values in patients with Cushing's disease. Clin Endocrinol (Oxf) (2014) 80(2):261–9. doi: 10.1111/cen.12259 PMC4231220 23746264

[20] Fleseriu M Auchus R Bancos I Ben-Shlomo A Bertherat J Biermasz NR. Consensus on diagnosis and management of Cushing's disease: a guideline update. Lancet Diabetes Endocrinol (2021) 9(12):847–75. doi: 10.1016/S2213-8587(21)00235-7 PMC8743006 34687601

[21] Zhang M Moore GA Jensen BP Begg EJ Bird PA. Determination of dexamethasone and dexamethasone sodium phosphate in human plasma and cochlear perilymph by liquid chromatography/tandem mass spectrometry. J Chromatogr B Analyt Technol BioMed Life Sci (2011) 879(1):17–24. doi: 10.1016/j.jchromb.2010.11.003 21112257

[22] Hawley JM Owen LJ Debono M Newell-Price J Keevil BG. Development of a rapid liquid chromatography tandem mass spectrometry method for the quantitation of serum dexamethasone and its clinical verification. Ann Clin Biochem (2018) 55(6):665–72. doi: 10.1177/0004563218766566 29534610

[23] Issa BG Hanna FWF Fryer AA Ensah G Ebere I Marshall D. The utility of salivary cortisone in the overnight dexamethasone suppression test in adrenal incidentalomas. J Clin Endocrinol Metab (2023) 25(5):698–700. doi: 10.1210/clinem/dgad242 37155577

[24] Raff H. Late Night Salivary Cortisol in the diagnosis of neoplastic hypercortisolism (including cyclic Cushing's syndrome). Pituitary (2022) 25(5):698–700. doi: 10.1007/s11102-022-01214-2 35334030

[25] Bansal V El Asmar N Selman WR Arafah BM. Pitfalls in the diagnosis and management of Cushing's syndrome. Neurosurg Focus (2015) 38(2):E4. doi: 10.3171/2014.11.FOCUS14704 25639322

[26] Liu H Bravata DM Cabaccan J Raff H Ryzen E. Elevated late-night salivary cortisol levels in elderly male type 2 diabetic veterans. Clin Endocrinol (Oxf) (2005) 63(6):642–9. doi: 10.1111/j.1365-2265.2005.02395.x 16343098

[27] Ma X Lian QQ Dong Q Ge RS. Environmental inhibitors of 11beta-hydroxysteroid dehydrogenase type 2. Toxicology (2011) 285(3):83–9. doi: 10.1016/j.tox.2011.04.007 21515335

[28] Lindsay JR Jonklaas J Oldfield EH Nieman LK. Cushing's syndrome during pregnancy: personal experience and review of the literature. J Clin Endocrinol Metab (2005) 90(5):3077–83. doi: 10.1210/jc.2004-2361 15705919

[29] Lopes LM Francisco RP Galletta MA Bronstein MD. Determination of nighttime salivary cortisol during pregnancy: comparison with values in non-pregnancy and Cushing's disease. Pituitary (2016) 19(1):30–8. doi: 10.1007/s11102-015-0680-3 26346684

[30] Alwani RA Schmit Jongbloed LW de Jong FH van der Lely AJ de Herder WW Feelders RA. Differentiating between Cushing's disease and pseudo-Cushing's syndrome: comparison of four tests. Eur J Endocrinol (2014) 170(4):477–86. doi: 10.1530/EJE-13-0702 24394725

[31] Stroud A Zhang J McCormack A. Diagnosing Cushing's disease in the context of chronic kidney disease: a case report and literature review. Eur J Endocrinol (2019) 181(4):K29–k35. doi: 10.1530/EJE-19-0326 31382242

[32] Buchfelder M Nistor R Fahlbusch R Huk WJ. The accuracy of CT and MR evaluation of the sella turcica for detection of adrenocorticotropic hormone-secreting adenomas in Cushing disease. AJNR Am J Neuroradiol (1993) 14(5):1183–90. doi: 10.1210/jc.2002-021438 PMC8332751 8237701

[33] Patronas N Bulakbasi N Stratakis CA Lafferty A Oldfield EH Doppman J. Spoiled gradient recalled acquisition in the steady state technique is superior to conventional postcontrast spin echo technique for magnetic resonance imaging detection of adrenocorticotropin-secreting pituitary tumors. J Clin Endocrinol Metab (2003) 88(4):1565–9. doi: 10.1210/jc.2002-021438 12679440

[34] Vassiliadi DA Mourelatos P Kratimenos T Tsagarakis S. Inferior petrosal sinus sampling in Cushing's syndrome: usefulness and pitfalls. Endocrine (2021) 73(3):530–9. doi: 10.1007/s12020-021-02764-4 34080096

[35] Wester VL Reincke M Koper JW van den Akker ELT Manenschijn L Berr CM. Scalp hair cortisol for diagnosis of Cushing's syndrome. Eur J Endocrinol (2017) 176(6):695–703. doi: 10.1530/EJE-16-0873 28289104

[36] Staufenbiel SM Penninx BW Spijker AT Elzinga BM van Rossum EF. Hair cortisol, stress exposure, and mental health in humans: a systematic review. Psychoneuroendocrinology (2013) 38(8):1220–35. doi: 10.1016/j.psyneuen.2012.11.015 23253896

[37] Short SJ Stalder T Marceau K Entringer S Moog NK Shirtcliff EA. Correspondence between hair cortisol concentrations and 30-day integrated daily salivary and weekly urinary cortisol measures. Psychoneuroendocrinology (2016) 71:12–8. doi: 10.1016/j.psyneuen.2016.05.007 PMC4955743 27235635

[38] van der Valk E Abawi O Mohseni M Abdelmoumen A Wester V van der Voorn B. Cross-sectional relation of long-term glucocorticoids in hair with anthropometric measurements and their possible determinants: A systematic review and meta-analysis. Obes Rev (2022) 23(3):e13376. doi: 10.1111/obr.13376 34811866 PMC9285618

[39] Brossaud J Charret L De Angeli D Haissaguerre M Ferriere A Puerto M. Hair cortisol and cortisone measurements for the diagnosis of overt and mild Cushing's syndrome. Eur J Endocrinol (2021) 184(3):445–54. doi: 10.1530/EJE-20-1127 33449913

[40] Manenschijn L Koper JW van den Akker EL de Heide LJ Geerdink EA de Jong FH. A novel tool in the diagnosis and follow-up of (cyclic) Cushing's syndrome: measurement of long-term cortisol in scalp hair. J Clin Endocrinol Metab (2012) 97(10):E1836–43. doi: 10.1210/jc.2012-1852 22844063

[41] Fassnacht M Arlt W Bancos I Dralle H Newell-Price J Sahdev A. Management of adrenal incidentalomas: European Society of Endocrinology Clinical Practice Guideline in collaboration with the European Network for the Study of Adrenal Tumors. Eur J Endocrinol (2016) 175(2):G1–G34. doi: 10.1530/EJE-16-0467 27390021

[42] Reincke M. Subclinical cushing's syndrome. Endocrinol Metab Clin North Am (2000) 29(1):43–56. doi: 10.1016/S0889-8529(05)70115-8 10732263

[43] Terzolo M Bovio S Reimondo G Pia A Osella G Borretta G. Subclinical Cushing's syndrome in adrenal incidentalomas. Endocrinol Metab Clin North Am (2005) 34(2):423–39. doi: 10.1016/j.ecl.2005.01.008 15850851

[44] Li S Wu Y Du X Li X Patel A Peterson ED. Rational and design of a stepped-wedge cluster randomized trial evaluating quality improvement initiative for reducing cardiovascular events among patients with acute coronary syndromes in resource-constrained hospitals in China. Am Heart J (2015) 169(3):349–55. doi: 10.1016/j.ahj.2014.12.005 25728724

[45] Sbardella E Minnetti M D'Aluisio D Rizza L Di Giorgio MR Vinci F. Cardiovascular features of possible autonomous cortisol secretion in patients with adrenal incidentalomas. Eur J Endocrinol (2018) 178(5):501–11. doi: 10.1530/EJE-17-0986 29510982

[46] Morelli V Reimondo G Giordano R Della Casa S Policola C Palmieri S. Long-term follow-up in adrenal incidentalomas: an Italian multicenter study. J Clin Endocrinol Metab (2014) 99(3):827–34. doi: 10.1210/jc.2013-3527 24423350

[47] Terzolo M Pia A Alì A Osella G Reimondo G Bovio S. Adrenal incidentaloma: a new cause of the metabolic syndrome? J Clin Endocrinol Metab (2002) 87(3):998–1003. doi: 10.1210/jcem.87.3.8277 11889151

[48] Patrova J Kjellman M Wahrenberg H Falhammar H. Increased mortality in patients with adrenal incidentalomas and autonomous cortisol secretion: a 13-year retrospective study from one center. Endocrine (2017) 58(2):267–75. doi: 10.1007/s12020-017-1400-8 28887710

[49] Di Dalmazi G Vicennati V Garelli S Casadio E Rinaldi E Giampalma E. Cardiovascular events and mortality in patients with adrenal incidentalomas that are either non-secreting or associated with intermediate phenotype or subclinical Cushing's syndrome: a 15-year retrospective study. Lancet Diabetes Endocrinol (2014) 2(5):396–405. doi: 10.1016/S2213-8587(13)70211-0 24795253

[50] Debono M Bradburn M Bull M Harrison B Ross RJ Newell-Price J. Cortisol as a marker for increased mortality in patients with incidental adrenocortical adenomas. J Clin Endocrinol Metab (2014) 99(12):4462–70. doi: 10.1210/jc.2014-3007 PMC4255126 25238207

[51] Deutschbein T Reimondo G Di Dalmazi G Bancos I Patrova J Vassiliadi DA. Age-dependent and sex-dependent disparity in mortality in patients with adrenal incidentalomas and autonomous cortisol secretion: an international, retrospective, cohort study. Lancet Diabetes Endocrinol (2022) 10(7):499–508. doi: 10.1016/S2213-8587(22)00100-0 35533704 PMC9679334

[52] Fassnacht M Dekkers OM Else T Baudin E Berruti A de Krijger R. European Society of Endocrinology Clinical Practice Guidelines on the management of adrenocortical carcinoma in adults, in collaboration with the European Network for the Study of Adrenal Tumors. Eur J Endocrinol (2018) 179(4):G1–G46. doi: 10.1530/EJE-18-0608 30299884

[53] Reimondo G Puglisi S Pia A Terzolo M. Autonomous hypercortisolism: definition and clinical implications. Minerva Endocrinol (2019) 44(1):33–42. doi: 10.23736/S0391-1977.18.02884-5 29963828

[54] Puglisi S Leporati M Amante E Parisi A Pia AR Berchialla P. Limited role of hair cortisol and cortisone measurement for detecting cortisol autonomy in patients with adrenal incidentalomas. Front Endocrinol (Lausanne) (2022) 13:833514. doi: 10.3389/fendo.2022.833514 35222288 PMC8863572

[55] Thomson S Koren G Fraser LA Rieder M Friedman TC Van Uum SH. Hair analysis provides a historical record of cortisol levels in Cushing's syndrome. Exp Clin Endocrinol Diabetes (2010) 118(2):133–8. doi: 10.1055/s-0029-1220771 PMC2945912 19609841

[56] van Boven E Massolt ET van Rossum EFC Kiewiet-Kemper RM. Spontaneous remission of unidentified Cushing's disease revealed by hair cortisol analysis. Neth J Med (2020) 78(5):297–9. 33093257

[57] Vassiliadi DA Tsagarakis S. DIAGNOSIS OF ENDOCRINE DISEASE: The role of the desmopressin test in the diagnosis and follow-up of Cushing's syndrome. Eur J Endocrinol (2018) 178(5):R201–R14. doi: 10.1530/EJE-18-0007 29472379

[58] Robinson AG. DDAVP in the treatment of central diabetes insipidus. N Engl J Med (1976) 294(10):507–11. doi: 10.1056/NEJM197603042941001 1250255

[59] Malerbi DA Mendonca BB Liberman B Toledo SP Corradini MC Cunha-Neto MB. The desmopressin stimulation test in the differential diagnosis of Cushing's syndrome. Clin Endocrinol (Oxf) (1993) 38(5):463–72. doi: 10.1111/j.1365-2265.1993.tb00341.x 8330442

[60] Tsagarakis S Tsigos C Vasiliou V Tsiotra P Kaskarelis J Sotiropoulou C. The desmopressin and combined CRH-desmopressin tests in the differential diagnosis of ACTH-dependent Cushing's syndrome: constraints imposed by the expression of V2 vasopressin receptors in tumors with ectopic ACTH secretion. J Clin Endocrinol Metab (2002) 87(4):1646–53. doi: 10.1210/jcem.87.4.8358 11932296

[61] Dahia PL Grossman AB. The molecular pathogenesis of corticotroph tumors. Endocr Rev (1999) 20(2):136–55. doi: 10.1210/edrv.20.2.0358 10204115

[62] Pinelli S Barbot M Scaroni C Ceccato F. Second-line tests in the diagnosis of adrenocorticotropic hormone-dependent hypercortisolism. Ann Lab Med (2021) 41(6):521–31. doi: 10.3343/alm.2021.41.6.521 PMC8203434 34108279

[63] Mondin A Barbot M Voltan G Tizianel I Vedolin CK Mazzeo P. Second-line tests in the differential diagnosis of neoplastic and non-neoplastic hypercortisolism: a systematic review and meta-analysis. J Endocrinol Invest (2023). doi: 10.1007/s40618-023-02099-z PMC10514124 37079177

[64] Moro M Putignano P Losa M Invitti C Maraschini C Cavagnini F. The desmopressin test in the differential diagnosis between Cushing's disease and pseudo-Cushing states. J Clin Endocrinol Metab (2000) 85(10):3569–74. doi: 10.1210/jcem.85.10.6862 11061503

[65] Pecori Giraldi F Pivonello R Ambrogio AG De Martino MC De Martin M Scacchi M. The dexamethasone-suppressed corticotropin-releasing hormone stimulation test and the desmopressin test to distinguish Cushing's syndrome from pseudo-Cushing's states. Clin Endocrinol (Oxf) (2007) 66(2):251–7. doi: 10.1111/j.1365-2265.2006.02717.x 17223996

[66] Tirabassi G Faloia E Papa R Furlani G Boscaro M Arnaldi G. Use of the desmopressin test in the differential diagnosis of pseudo-Cushing state from Cushing's disease. J Clin Endocrinol Metab (2010) 95(3):1115–22. doi: 10.1210/jc.2009-1146 20080839

[67] Rollin GA Costenaro F Gerchman F Rodrigues TC Czepielewski MA. Evaluation of the DDAVP test in the diagnosis of Cushing's Disease. Clin Endocrinol (Oxf) (2015) 82(6):793–800. doi: 10.1111/cen.12661 25376361

[68] Sakai Y Horiba N Tozawa F Sakai K Kuwayama A Demura H. Desmopressin stimulation test for diagnosis of ACTH-dependent Cushing's syndrome. Endocr J (1997) 44(5):687–95. doi: 10.1507/endocrj.44.687 9466324

[69] Tsagarakis S Vasiliou V Kokkoris P Stavropoulos G Thalassinos N. Assessment of cortisol and ACTH responses to the desmopressin test in patients with Cushing's syndrome and simple obesity. Clin Endocrinol (Oxf) (1999) 51(4):473–7. doi: 10.1046/j.1365-2265.1999.00830.x 10583315

[70] Suda T Kageyama K Nigawara T Sakihara S. Evaluation of diagnostic tests for ACTH-dependent Cushing's syndrome. Endocr J (2009) 56(3):469–76. doi: 10.1507/endocrj.K08E-353 19225213

[71] Ceccato F Barbot M Mondin A Boscaro M Fleseriu M Scaroni C. Dynamic testing for differential diagnosis of ACTH-dependent cushing syndrome: A systematic review and meta-analysis. J Clin Endocrinol Metab (2023) 108(5):e178–e88. doi: 10.1210/clinem/dgac686 36453141

[72] Valassi E Swearingen B Lee H Nachtigall LB Donoho DA Klibanski A. Concomitant medication use can confound interpretation of the combined dexamethasone-corticotropin releasing hormone test in Cushing's syndrome. J Clin Endocrinol Metab (2009) 94(12):4851–9. doi: 10.1210/jc.2009-1500 PMC2795659 19850679

[73] Alexandraki KI Kaltsas GA Isidori AM Storr HL Afshar F Sabin I. Long-term remission and recurrence rates in Cushing's disease: predictive factors in a single-centre study. Eur J Endocrinol (2013) 168(4):639–48. doi: 10.1530/EJE-12-0921 23371975

[74] Chen JC Amar AP Choi S Singer P Couldwell WT Weiss MH. Transsphenoidal microsurgical treatment of Cushing disease: postoperative assessment of surgical efficacy by application of an overnight low-dose dexamethasone suppression test. J Neurosurg (2003) 98(5):967–73. doi: 10.3171/jns.2003.98.5.0967 12744355

[75] Colombo P Dall'Asta C Barbetta L Re T Passini E Faglia G. Usefulness of the desmopressin test in the postoperative evaluation of patients with Cushing's disease. Eur J Endocrinol (2000) 143(2):227–34. doi: 10.1530/eje.0.1430227 10913942

[76] Romanholi DJ Machado MC Pereira CC Danilovic DS Pereira MA Cescato VA. Role for postoperative cortisol response to desmopressin in predicting the risk for recurrent Cushing's disease. Clin Endocrinol (Oxf) (2008) 69(1):117–22. doi: 10.1111/j.1365-2265.2007.03168.x 18182093

[77] Valéro R Vallette-Kasic S Conte-Devolx B Jaquet P Brue T. The desmopressin test as a predictive factor of outcome after pituitary surgery for Cushing's disease. Eur J Endocrinol (2004) 151(6):727–33. doi: 10.1530/eje.0.1510727 15588239

[78] Vassiliadi DA Balomenaki M Asimakopoulou A Botoula E Tzanela M Tsagarakis S. The desmopressin test predicts better than basal cortisol the long-term surgical outcome of cushing's disease. J Clin Endocrinol Metab (2016) 101(12):4878–85. doi: 10.1210/jc.2016-2799 27662440

[79] Ambrosi B Malavazos AE Passeri E Dall'Asta C. Desmopressin test may predict the risk of recurrence in Cushing's disease. Clin Endocrinol (Oxf) (2009) 70(5):811. doi: 10.1111/j.1365-2265.2008.03406.x 18771561

[80] Bou Khalil R Baudry C Guignat L Carrasco C Guibourdenche J Gaillard S. Sequential hormonal changes in 21 patients with recurrent Cushing's disease after successful pituitary surgery. Eur J Endocrinol (2011) 165(5):729–37. doi: 10.1530/EJE-11-0424 21885674

[81] Ambrogio AG Andrioli M De Martin M Cavagnini F Pecori Giraldi F. Usefulness of desmopressin testing to predict relapse during long-term follow-up in patients in remission from Cushing's disease. Endocr Connect (2017) 6(8):791–9. doi: 10.1530/EC-17-0292 PMC5682421 29018154

[82] Hayes AR Grossman AB. The ectopic adrenocorticotropic hormone syndrome: rarely easy, always challenging. Endocrinol Metab Clin North Am (2018) 47(2):409–25. doi: 10.1016/j.ecl.2018.01.005 29754641

[83] Lindsay JR Nieman LK. Differential diagnosis and imaging in Cushing's syndrome. Endocrinol Metab Clin North Am (2005) 34(2):403–21. doi: 10.1016/j.ecl.2005.01.009 15850850

[84] Pecori Giraldi F Cavallo LM Tortora F Pivonello R Colao A Cappabianca P. The role of inferior petrosal sinus sampling in ACTH-dependent Cushing's syndrome: review and joint opinion statement by members of the Italian Society for Endocrinology, Italian Society for Neurosurgery, and Italian Society for Neuroradiology. Neurosurg Focus (2015) 38(2):E5. doi: 10.3171/2014.11.FOCUS14766 25639323

[85] Aron DC Raff H Findling JW. Effectiveness versus efficacy: the limited value in clinical practice of high dose dexamethasone suppression testing in the differential diagnosis of adrenocorticotropin-dependent Cushing's syndrome. J Clin Endocrinol Metab (1997) 82(6):1780–5. doi: 10.1210/jc.82.6.1780 9177382

[86] Frete C Corcuff JB Kuhn E Salenave S Gaye D Young J. Non-invasive diagnostic strategy in ACTH-dependent Cushing's syndrome. J Clin Endocrinol Metab (2020) 105(10). doi: 10.1210/clinem/dgaa409 32594169

[87] Barbot M Trementino L Zilio M Ceccato F Albiger N Daniele A. Second-line tests in the differential diagnosis of ACTH-dependent Cushing's syndrome. Pituitary (2016) 19(5):488–95. doi: 10.1007/s11102-016-0729-y 27236452

[88] Ferrante E Barbot M Serban AL Ceccato F Carosi G Lizzul L. Indication to dynamic and invasive testing in Cushing's disease according to different neuroradiological findings. J Endocrinol Invest (2022) 45(3):629–37. doi: 10.1007/s40618-021-01695-1 PMC8850245 34699044

[89] Grasperge BJ Morgan TW Paddock CD Peterson KE Macaluso KR. Feeding by Amblyomma maculatum (Acari: Ixodidae) enhances Rickettsia parkeri (Rickettsiales: Rickettsiaceae) infection in the skin. J Med Entomol (2014) 51(4):855–63. doi: 10.1603/ME13248 PMC4214552 25118419

[90] Lee MD Young MG Fatterpekar GM. "The pituitary within GRASP" - golden-angle radial sparse parallel dynamic MRI technique and applications to the pituitary gland. Semin Ultrasound CT MR (2021) 42(3):307–15. doi: 10.1053/j.sult.2021.04.007 34147165

[91] Castle-Kirszbaum M Amukotuwa S Fuller P Goldschlager T Gonzalvo A Kam J. MRI for cushing disease: A systematic review. AJNR Am J Neuroradiol (2023) 44(3):311–6. doi: 10.3174/ajnr.A7789 PMC10187804 36759141

[92] Newell-Price J Trainer P Besser M Grossman A. The diagnosis and differential diagnosis of Cushing's syndrome and pseudo-Cushing's states. Endocr Rev (1998) 19(5):647–72. doi: 10.1210/edrv.19.5.0346 9793762

[93] Yamada S Fukuhara N Nishioka H Takeshita A Inoshita N Ito J. Surgical management and outcomes in patients with Cushing disease with negative pituitary magnetic resonance imaging. World Neurosurg (2012) 77(3-4):525–32. doi: 10.1016/j.wneu.2011.06.033 22120352

[94] Chittiboina P Montgomery BK Millo C Herscovitch P Lonser RR. High-resolution(18)F-fluorodeoxyglucose positron emission tomography and magnetic resonance imaging for pituitary adenoma detection in Cushing disease. J Neurosurg (2015) 122(4):791–7. doi: 10.3171/2014.10.JNS14911 PMC5091074 25479121

[95] Bashari WA Gillett D MacFarlane J Powlson AS Kolias AG Mannion R. Modern imaging in Cushing's disease. Pituitary (2022) 25(5):709–12. doi: 10.1007/s11102-022-01236-w PMC9587975 35666391

[96] Berkmann S Roethlisberger M Mueller B Christ-Crain M Mariani L Nitzsche E. Selective resection of cushing microadenoma guided by preoperative hybrid 18-fluoroethyl-L-tyrosine and 11-C-methionine PET/MRI. Pituitary (2021) 24(6):878–86. doi: 10.1007/s11102-021-01160-5 34155554

[97] Isidori AM Sbardella E Zatelli MC Boschetti M Vitale G Colao A. Conventional and nuclear medicine imaging in ectopic cushing's syndrome: A systematic review. J Clin Endocrinol Metab (2015) 100(9):3231–44. doi: 10.1210/JC.2015-1589 PMC4570166 26158607

[98] Wannachalee T Turcu AF Bancos I Habra MA Avram AM Chuang HH. The clinical impact of [(68) Ga]-DOTATATE PET/CT for the diagnosis and management of ectopic adrenocorticotropic hormone - secreting tumours. Clin Endocrinol (Oxf) (2019) 91(2):288–94. doi: 10.1111/cen.14008 PMC6689243 31066920

[99] Walia R Gupta R Bhansali A Pivonello R Kumar R Singh H. Molecular imaging targeting corticotropin-releasing hormone receptor for corticotropinoma: A changing paradigm. J Clin Endocrinol Metab (2021) 106(4):e1816–e26. doi: 10.1210/clinem/dgaa755 33079979

[100] Koong K Preda V Jian A Liquet-Weiland B Di Ieva A. Application of artificial intelligence and radiomics in pituitary neuroendocrine and sellar tumors: a quantitative and qualitative synthesis. Neuroradiology (2022) 64(4):647–68. doi: 10.1007/s00234-021-02845-1 34839380

[101] Di Ieva A Russo C Liu S Jian A Bai MY Qian Y. Application of deep learning for automatic segmentation of brain tumors on magnetic resonance imaging: a heuristic approach in the clinical scenario. Neuroradiology (2021) 63(8):1253–62. doi: 10.1007/s00234-021-02649-3 33501512

[102] Zoli M Staartjes VE Guaraldi F Friso F Rustici A Asioli S. Machine learning-based prediction of outcomes of the endoscopic endonasal approach in Cushing disease: is the future coming? Neurosurg Focus (2020) 48(6):E5. doi: 10.3171/2020.3.FOCUS2060 32480364

[103] Zhang W Sun M Fan Y Wang H Feng M Zhou S. Machine learning in preoperative prediction of postoperative immediate remission of histology-positive cushing's disease. Front Endocrinol (Lausanne) (2021) 12:635795. doi: 10.3389/fendo.2021.635795 33737912 PMC7961560

[104] Fan Y Li Y Bao X Zhu H Lu L Yao Y. Development of machine learning models for predicting postoperative delayed remission in patients with cushing's disease. J Clin Endocrinol Metab (2021) 106(1):e217–e31. doi: 10.1210/clinem/dgaa698 33000120

[105] Elmohr MM Fuentes D Habra MA Bhosale PR Qayyum AA Gates E. Machine learning-based texture analysis for differentiation of large adrenal cortical tumours on CT. Clin Radiol (2019) 74(10):818.e1–e7. doi: 10.1016/j.crad.2019.06.021 31362884

[106] Yu H Parakh A Blake M McDermott S. Texture analysis as a radiomic marker for differentiating benign from malignant adrenal tumors. J Comput Assist Tomogr (2020) 44(5):766–71. doi: 10.1097/RCT.0000000000001051 32842071

[107] Greff MJE Levine JM Abuzgaia AM Elzagallaai AA Rieder MJ van Uum SHM. Hair cortisol analysis: An update on methodological considerations and clinical applications. Clin Biochem (2019) 63:1–9. doi: 10.1016/j.clinbiochem.2018.09.010 30261181

[108] Saluti G Ricci M Castellani F Colagrande MN Di Bari G Vulpiani MP. Determination of hair cortisol in horses: comparison of immunoassay vs LC-HRMS/MS. Anal Bioanal Chem (2022) 414(28):8093–105. doi: 10.1007/s00216-022-04343-6 PMC9613578 36136115

[109] Noppe G de Rijke YB Dorst K van den Akker EL van Rossum EF. LC-MS/MS-based method for long-term steroid profiling in human scalp hair. Clin Endocrinol (Oxf) (2015) 83(2):162–6. doi: 10.1111/cen.12781 25823708

[110] Nugent CA Warner HR Dunn JT Tyler FH. PROBABILITY THEORY IN THE DIAGNOSIS OF CUSHING'S SYNDROME. J Clin Endocrinol Metab (1964) 24:621–7. doi: 10.1210/jcem-24-7-621 14212081

[111] León-Justel A Madrazo-Atutxa A Alvarez-Rios AI Infantes-Fontán R Garcia-Arnés JA Lillo-Muñoz JA. A probabilistic model for cushing's syndrome screening in at-risk populations: A prospective multicenter study. J Clin Endocrinol Metab (2016) 101(10):3747–54. doi: 10.1210/jc.2016-1673 27490917

[112] Parasiliti-Caprino M Bioletto F Frigerio T D'Angelo V Ceccato F Ferrau F. A new clinical model to estimate the pre-test probability of cushing's syndrome: the cushing score. Front Endocrinol (Lausanne) (2021) 12:747549. doi: 10.3389/fendo.2021.747549 34675882 PMC8524092

[113] Braun LT Riester A Oßwald-Kopp A Fazel J Rubinstein G Bidlingmaier M. Toward a diagnostic score in cushing's syndrome. Front Endocrinol (Lausanne) (2019) 10:766. doi: 10.3389/fendo.2019.00766 31787931 PMC6856055

[114] Lam-Chung CE Cuevas-Ramos D. The promising role of risk scoring system for Cushing syndrome: Time to reconsider current screening recommendations. Front Endocrinol (Lausanne) (2022) 13:1075785. doi: 10.3389/fendo.2022.1075785 36482998 PMC9725023

